# Comparison of influenza surveillance systems in Australia, China, Malaysia and expert recommendations for influenza control

**DOI:** 10.1186/s12889-021-11765-x

**Published:** 2021-09-26

**Authors:** Clotilde El Guerche-Séblain, Thierry Rigoine De Fougerolles, Kim Sampson, Lance Jennings, Paul Van Buynder, Yuelong Shu, Zamberi Sekawi, Leo Yee-Sin, Tony Walls, Olivier Vitoux, J. Kevin Yin, Ada Wong, Francois Schellevis, Philippe Vanhems

**Affiliations:** 1 Global Medical Evidence Generation (MEG) Lead, Influenza Sanofi Pasteur, Medical Influenza Franchise, Sanofi-Aventis (Singapore) Pte. Ltd. 38, Beach Road, #18-11, South Beach Tower, Sanofi Pasteur, Singapore, Singapore; 2 University Claude Bernard Lyon 1, Lyon, France; 3 CVA, Paris, France; 4 Asia-Pacific Alliance for the Control of Influenza (APACI), Melbourne, Australia; 5 Australian Immunisation Coalition, Melbourne, Australia; 6 University of Otago, Christchurch, New Zealand; 7 Canterbury Health Laboratories, Christchurch, New Zealand; 8 Department of Public Health, Griffith University, Griffith, Victoria Australia; 9 School of Public Health (Shenzhen), Sun Yat-sen University, Shenzhen, China; 10 Chair Malaysia Influenza Working Group (MIWG), Universiti Putra, Seri Kembangan, Malaysia; 11 National Center for Infectious Diseases (NCID), Singapore, Singapore; 12 Paediatric Society of New Zealand Infection and Immunization Special Interest Group, Christchurch, New Zealand; 13 Global Medical Affairs, Sanofi Pasteur, Singapore, Singapore; 14 Faculty of Medicine and Health, The University of Sydney, Sydney, Australia; 15 Public Affairs, Sanofi Pasteur, Singapore, Singapore; 16 Netherlands Institute for Health Services Research, Utrecht, The Netherlands; 17 Department of General Practice, Amsterdam Public Health Research Institute, Amsterdam University Medical Centres, Amsterdam, The Netherlands; 18 Unité d’Hygiène, Epidémiologie, Infectiovigilance et Prévention, Hospices Civils de Lyon, Lyon, France; 19 PHE3ID, Centre International de Recherche en Infectiologie, Institut National de la Santé et de la Recherche Médicale U1111, CNRS Unité Mixte de Recherche 5308, École Nationale Supérieure de Lyon, Université Claude Bernard Lyon 1, Lyon, France

**Keywords:** Comparative study, Western Pacific region, Framework, Influenza, Surveillance system, WHO guideline adherence, Expert recommendations

## Abstract

**Background:**

The Western Pacific Region (WPR) is exposed each year to seasonal influenza and is often the source of new influenza virus variants and novel pathogen emergence. National influenza surveillance systems play a critical role in detecting emerging viruses, monitoring influenza epidemics, improving public disease awareness and promoting pandemic preparedness, but vary widely across WPR countries. The aim of this study is to improve existing influenza surveillance systems by systematically comparing selected WPR influenza surveillance systems.

**Methods:**

Three national influenza surveillance systems with different levels of development (Australia, China and Malaysia) were compared and their adherence to World Health Organization (WHO) guidance was evaluated using a structured framework previously tested in several European countries consisting of seven surveillance sub-systems, 19 comparable outcomes and five evaluation criteria. Based on the results, experts from the Asia-Pacific Alliance for the Control of Influenza (APACI) issued recommendations for the improvement of existing surveillance systems.

**Results:**

Australia demonstrated the broadest scope of influenza surveillance followed by China and Malaysia. In Australia, surveillance tools covered all sub-systems. In China, surveillance did not cover non-medically attended respiratory events, primary care consultations, and excess mortality modelling. In Malaysia, surveillance consisted of primary care and hospital sentinel schemes. There were disparities between the countries across the 5 evaluation criteria, particularly regarding data granularity from health authorities, information on data representativeness, and data communication, especially the absence of publicly available influenza epidemiological reports in Malaysia. This dual approach describing the scope of surveillance and evaluating the adherence to WHO guidance enabled APACI experts to make a number of recommendations for each country that included but were not limited to introducing new surveillance tools, broadening the use of specific existing surveillance tools, collecting and sharing data on virus characteristics, developing immunization status registries, and improving public health communication.

**Conclusions:**

Influenza monitoring in Australia, China, and Malaysia could benefit from the expansion of existing surveillance sentinel schemes, the broadened use of laboratory confirmation and the introduction of excess-mortality modelling. The results from the evaluation can be used as a basis to support expert recommendations and to enhance influenza surveillance capabilities.

**Supplementary Information:**

The online version contains supplementary material available at 10.1186/s12889-021-11765-x.

## Background

The Western Pacific Region (WPR), which covers 37 countries with more than one quarter of the world’s population, is particularly notorious for influenza virus mutations and the emergence of new respiratory pathogens due to the large human and animal populations and their interaction [1–3]. Surveillance systems are essential not only to describe the epidemiology and burden of seasonal influenza epidemics, but also to provide isolates for the characterisation of influenza viruses for vaccine strain selection and to identify new pathogens with pandemic potential. These systems are also used by health authorities to measure the impact of national vaccination programmes and to monitor antiviral drug resistance [4, 5]. Although extensive influenza epidemiological data exist for Europe and North America, much less data are available for the WPR, especially if data from China is excluded [6]. In terms of hospitalization and mortality, the burden of influenza in some tropical and subtropical zones in the WPR appears to be similar to that in temperate countries across the world, although data are lacking for many WPR countries [7].

The World Health Organization (WHO) Global Influenza Surveillance and Response System (GISRS) has played a critical role in developing our current understanding of influenza virus circulation in the WPR, through a network of WHO National Influenza Centres (NICs), Collaborating Centres (WHO CCs) and reference laboratories [8–10]. In the Global Influenza Strategy 2019–2030, WHO highlights the importance of strengthening global influenza surveillance, monitoring and data utilization [11], consistently with the recommendations of international experts in the region [6]. In WHO Influenza Vaccine Post-Introduction Evaluation framework, influenza surveillance is one of the key components of successful national influenza immunisation programmes to monitor the disease burden associated with influenza epidemics, best define the timing of the vaccination campaign and evaluate the impact of immunization strategies [12]. Yet, the proportion of WPR countries with a national seasonal influenza vaccination programme is considerably lower (26%) than in Europe and North America (76%), Central and South America (90%), and the Middle East (62%) [13–15]. This underutilization of influenza vaccination as a public health tool translates into low Vaccine Coverage Rates (VCR) across Asia, as recently highlighted in a review reporting a median uptake of 14.9% among the general population and 37.3% among high-risk groups, falling short from the WHO 75% target despite wide disparities across countries [16]. Improving national surveillance systems to produce accurate estimates of the overall burden and severity of influenza is instrumental to inform prevention policy-making and feed public awareness that will ultimately contribute to higher VCR in the region [17–19].

Although WHO regularly evaluates all surveillance systems under the Pandemic Influenza Preparedness framework, there is no published structured comparison of national influenza surveillance systems across WPR countries. We applied a comparative framework developed and tested in several European countries [20], and based on WHO guidance [21, 22], to surveillance systems in selected WPR countries with the following three aims: (i) to describe and compare the main characteristics of the existing influenza surveillance systems; (ii) to evaluate adherence to WHO guidance; (iii) to formulate expert recommendations on possible enhancements to surveillance systems to effectively monitor influenza and ultimately inform national influenza control decisions.

## Methods

### Selection of countries

Australia, China, and Malaysia were selected as countries representing each of the three WHO transmission zones of the WPR, where each zone covers a geographical group of countries with similar influenza transmission patterns [23, 24]. Australia is mainly temperate and located in the Oceania, Melanesia, Polynesia influenza transmission zone. China includes tropical, subtropical, and temperate zones, and is part of the Eastern Asia influenza transmission zone. Malaysia is tropical in climate and part of South-East Asia influenza transmission zone. Furthermore, the three countries present different healthcare system structures and proportion of the population covered under their influenza immunisation programme (large [Australia], moderate [China], and limited [Malaysia]) [25–27].

### Sources of information

Publicly available resources on influenza surveillance produced by the health authorities of each country were screened in English and local language. For Australia, information was collected from the surveillance system overview and fortnightly influenza epidemiological reports published by the Department of Health (DOH) and complemented by a search of epidemiological reports published at state level [28–30]. For China, information sources included the influenza surveillance protocol from the Chinese Center for Disease Control and Prevention (CCDC), the weekly influenza reports from the Chinese National Influenza Center (CNIC) and the monthly reports on infectious diseases from the National Health Commission [31–33]. For Malaysia, in the absence of publicly available epidemiological influenza reports, the Malaysia Influenza Surveillance Protocol published by the Ministry of Health (MOH) was used [34].

### Evaluation framework

The scope of surveillance systems were compared across seven sub-systems: 1) Non-medically attended community surveillance; 2) Virological surveillance including subtyping of influenza viruses, genome sequencing capabilities and antiviral drug resistance; 3) Community surveillance covering the notification of laboratory-confirmed cases; 4) Outbreak surveillance to report suspected or laboratory-confirmed influenza clusters in close settings where the public is allowed and can interact, such as care homes, schools or prisons 5); Primary care syndromic surveillance corresponding to sentinel and non-sentinel General Practitioners (GP) schemes; 6) Secondary care syndromic surveillance corresponding to the monitoring of mild and severe outcomes in hospitals and 7) Mortality surveillance encompassing death notifications and excess death statistical modelling. These sub-systems are not mutually exclusive and most often closely intertwined, with for instance syndromic surveillance performed by sentinel GPs (sub system 5) and sentinel hospitals (sub-system 6) sending samples collected from patients with respectively ILI and SARI symptoms to laboratories for influenza typing or sub-typing (sub system 2). Across those seven sub-systems, a list of 19 comparable outcomes was used to compare the scope of surveillance following a scale of severity from non-medically attended events to deaths (Table 1) [20].

**Table 1:** Influenza surveillance seven sub-systems and nineteen comparative outcomes [20]

Surveillance sub-system	Outcome
1. Non-medically attended community surveillance	1.1. ARI/ILI cases and/or incidence rates
community surveillance	1.2. Proportion of ARI/ILI cases seeking care
2. Virological surveillance	2.1. ARI/ILI specimens for virus typing & subtyping
	2.2. ARI/ILI specimens for virus genome sequencing
	2.3. ARI/ILI specimens for antiviral drug resistance
3. Community surveillance	3.1. Notified biologically/laboratory-confirmed cases
4. Outbreak surveillance	4.1. ARI/ILI outbreaks in close settings
	4.2. Biologically/laboratory-confirmed outbreaks in close settings
5. Primary care syndromic surveillance	5.1. ARI/ILI GP visits and/or incidence rates
	5.2. Biologically/laboratory-confirmed GP visits and/or incidence rates
	5.3. Influenza-associated excess GP visits
	5.4. Influenza-associated excess work-loss cases
6. Hospital syndromic surveillance	6.1. ILI or biologically/laboratory-confirmed Emergency Department visits
	6.2. SARI/ILI hospital admissions
	6.3. Biologically/laboratory-confirmed hospital admissions
	6.4. Influenza-associated excess hospital admissions
	6.5. Biologically/laboratory-confirmed influenza ICU admissions
7. Mortality surveillance	7.1 Diagnosed or biologically/laboratory-confirmed influenza deaths
	7.2. Influenza-associated excess deaths

*GP* general practitioner, *ICU* intensive care unit, *ILI* influenza-like illness, *(S)ARI* (severe) acute respiratory illness;

The influenza surveillance tools used in each country were identified from the resources mentioned above and matched with the list of surveillance outcomes from the framework. Adherence to WHO guidance was assessed using five evaluation criteria: Granularity, Timing, Representativeness, Sampling Strategy and Communication inspired from WHO guidelines to measure and monitor the burden of seasonal influenza (Table 2) [21, 22].

**Table 2:** Evaluation of surveillance through five criteria and associated sub-criteria [20]

Criteria	Sub-criteria	WHO Guidance
Granularity	Age group	Recommended as a minimum: 0–1, 2–4, 5–14, 15–49, 50–64, 65+ years and ideally additional age strata for under 2 years including 0 to < 6 months, 6 month to < 1 year, 1 to < 2 years
	Gender	Where possible data should be extracted by gender
	Risk condition	Recommended as a minimum: pregnancy status & presence of chronic pre-existing medical illness(es): chronic respiratory disease, asthma, diabetes, chronic cardiac disease, chronic neurological or neuromuscular disease, haematological disorders, immunodeficiency (including Human Immunodeficiency Virus)
	Location	Considered as essential, especially for burden estimation for a given area based on data from sentinel sites
	Virology	Types and subtypes of viruses detected during the week
	Severity	Additional data to consider: signs and symptoms of illness & patient outcome (death, survival)
	Treatment	Exposure to influenza antiviral drugs during the last 14 days? If yes, name of antiviral
	Vaccination status	Additional data to consider: Seasonal influenza vaccination status and date of administration
Timing	Frequency	Epidemiological and virological data collected from the sentinel sites should be reported to the national health authorities on a weekly basis
	Time period	In temperate climate zones where influenza seasonality is well understood, data collection and reporting should occur at a minimum during the known influenza season and for a short period preceding and following the season
Representativeness	Geographical representativeness	National - sentinel sites should include patients that will appropriately represent the population
	Population representativeness	The population served by the sentinel site should be representative of the target age and socioeconomic groups in the population under surveillance
	Number of settings	There is no ideal number of sentinel sites in a country. Start small with one or a few sentinel sites and only expand if this functions well. Minimal information that should be presented in the weekly report includes number of sentinel sites reporting
	Proportion of facilities	Ideally the following analyses can be presented in an annual report: data from the monitoring of the system: proportion of sentinel sites reporting weekly to the national level; and if feasible, the proportion of sentinel sites regularly submitting specimens for laboratory testing
Sampling strategy	Surveillance type	Sentinel surveillance
	ARI/ILI definition	An acute respiratory infection with fever ≥38 °C and cough with onset within the last 10 days
	Sample collection	A systematic approach to case selection that does not leave the choice of cases to test or gather data from up to healthcare providers (other than to determine that the case meets the definition), and that covers different times of the day and different days of the week is likely to be the most pragmatic, while providing reasonably representative data
	Test type	Reverse transcriptase-polymerase chain reaction (RT-PCR) is the most sensitive method for detecting influenza virus and is the recommended influenza surveillance assay for laboratories
Communication	In annual report	Yearly surveillance report with surveillance and risk factor data should be produced
	In weekly report	Weekly surveillance reports should be produced and made accessible to relevant partners
	Delay in release	Reports should provide timely information on influenza activity and types of influenza viruses circulating
	Data can be extracted	Whenever feasible, such reports should be available to the public on the national surveillance website

*ARI* acute respiratory illness, *ILI* influenza-like illness, *RT-PCR* reverse transcriptase-polymerase chain reaction

^a^From WHO global epidemiological surveillance standards for influenza (2014) and WHO manual for estimating disease burden associated with seasonal influenza (2015)

For each surveillance sub-system, the surveillance tools used in each country were further detailed across the five evaluation criteria using sub-criteria. For instance, the granularity of a surveillance tool was described in terms of the availability of data stratified per age, per gender and at-risk condition among others.

The results from the comparison were reviewed by a panel of seven experts from the Asia-Pacific Alliance for the Control of Influenza (APACI), an organisation whose main aim is to reduce the burden of influenza by enhancing control measures and boosting pandemic preparedness in the Asia-Pacific region [35]. Capitalizing on the results from the comparative analysis and assessment of adherence to WHO guidance, the experts individually reviewed the evaluation of influenza surveillance systems, discussed identified gaps and shared recommendations in each country. Using an expert consensus approach during an online meeting, they proposed a selection of recommendations which represent the key elements to improve surveillance systems considering country specificities.

## Results

### Country comparison of the scope of national influenza surveillance systems (Table 3)

Australia showed the widest scope of influenza surveillance, covering all seven sub-systems with surveillance tools, and 16/19 outcomes, followed by China with 5/7 sub-systems and 11/19 outcomes and Malaysia with 4/7 sub-systems and 8/19 outcomes (Table 3). Details for the evaluation in each country of the seven sub-systems are available in the additional file.

**Table 3:** Overview of the comparative framework of influenza surveillance systems in Australia, China and Malaysia

Surveillance sub-system	Outcome	Australia	China	Malaysia
		Department of Health	Chinese Center for Disease Control and Prevention	Ministry of Health
1. Non-medically attended community surveillance	1.1. ARI/ILI cases and/or incidence rates	Web-survey (Info.flutracking.Net) & medical hotline (Healthdirect)	None	None
	1.2. Proportion of ARI/ILI cases seeking care		None	None
2. Virological surveillance	2.1. ARI / ILI specimens for virus typing & subtyping	Sentinel labs & 2 NIC	Sentinel labs (NISN) & 1 NIC	2 NIC
	2.2. ARI / ILI specimens for virus genome sequencing	1 WHO CC and H5 lab	1 WHO CC and H5 lab	None
	2.3. ARI / ILI specimens for antiviral drug resistance			
3. Community surveillance	3.1. Notified biologically/laboratory-confirmed cases	Sentinel labs, Mandatory disease notification (NNDSS)	Mandatory disease notification (NIDRS)	None
4. Outbreak surveillance	4.1. ARI/ILI outbreaks in close settings	Outbreak monitoring in Australian states	Outbreaks in close settings (CPHEMIS)	Unpublished
	4.2. Biologically /laboratory-confirmed outbreaks in close settings			Unpublished
5. Primary care syndromic surveillance	5.1. ARI/ILI GP visits and/or incidence rates	Sentinel GPs (ASPREN)	None	ILI sentinel clinics
	5.2. Biologically/ laboratory-confirmed GP visits and/or incidence rates		None	
	5.3. Influenza-associated excess GP visits	None	None	None
	5.4. Influenza-associated excess work loss cases	None	None	None
6. Hospital syndromic surveillance	6.1. ILI or biologically/laboratory-confirmed emergency department visits	ILI EDs (PHREDSS some states only - NSW)	ILI sentinel emergency departments (NISN)	None
	6.2. SARI/ILI hospital admissions	SARI sentinel hospitals (FluCAN & APSU)	SARI sentinel hospitals scheme	SARI sentinel hospitals
	6.3. Biologically/ laboratory-confirmed hospital admissions			
	6.4. Influenza-associated excess hospital admissions	None	None	None
	6.5 Biologically/ laboratory-confirmed ICU admissions	SARI sentinel hospitals (FluCAN & APSU)	SARI sentinel hospitals system	SARI sentinel hospitals
7. Mortality surveillance	7.1 Diagnosed or biologically/laboratory-confirmed influenza deaths	Mandatory disease notification (NNDSS)	Mandatory disease notification (NIDRS)	None
	7.2. Influenza-associated excess deaths	Influenza & pneumonia (some states only - NSW)	None	None

None = there are no surveillance tools covering this outcome; unpublished = these outcomes are covered by surveillance tools but the data are not published in the weekly or annual reports

*APSU* Australian Paediatric Surveillance Unit, *ARI* Acute Respiratory Infection, *ASPREN* Australian Sentinel Practices Research Network, *CPHEMIS* China Public Health Emergency Management Information System, *ED* Emergency Department, *FluCAN* The Influenza Complications Alert Network, *GP* General Practitioner, *ICU* Intensive Care Unit, *ILI* Influenza-Like Illness, *NIC* National Influenza Centre, *NIDRS* National Infectious Disease Reporting System, *NISN* National Influenza Surveillance Network, *NNDSS* National Notifiable Diseases Surveillance System, *NSW* New South Wales, *PHREDSS* Public Health Rapid, Emergency, Disease and Syndromic Surveillance, *SARI* Severe Acute Respiratory Infection, *WHO CC* World Health Organization Collaborating Centre

### Influenza surveillance systems in each country and identification of gaps

#### Australia

**Fig. 1:**
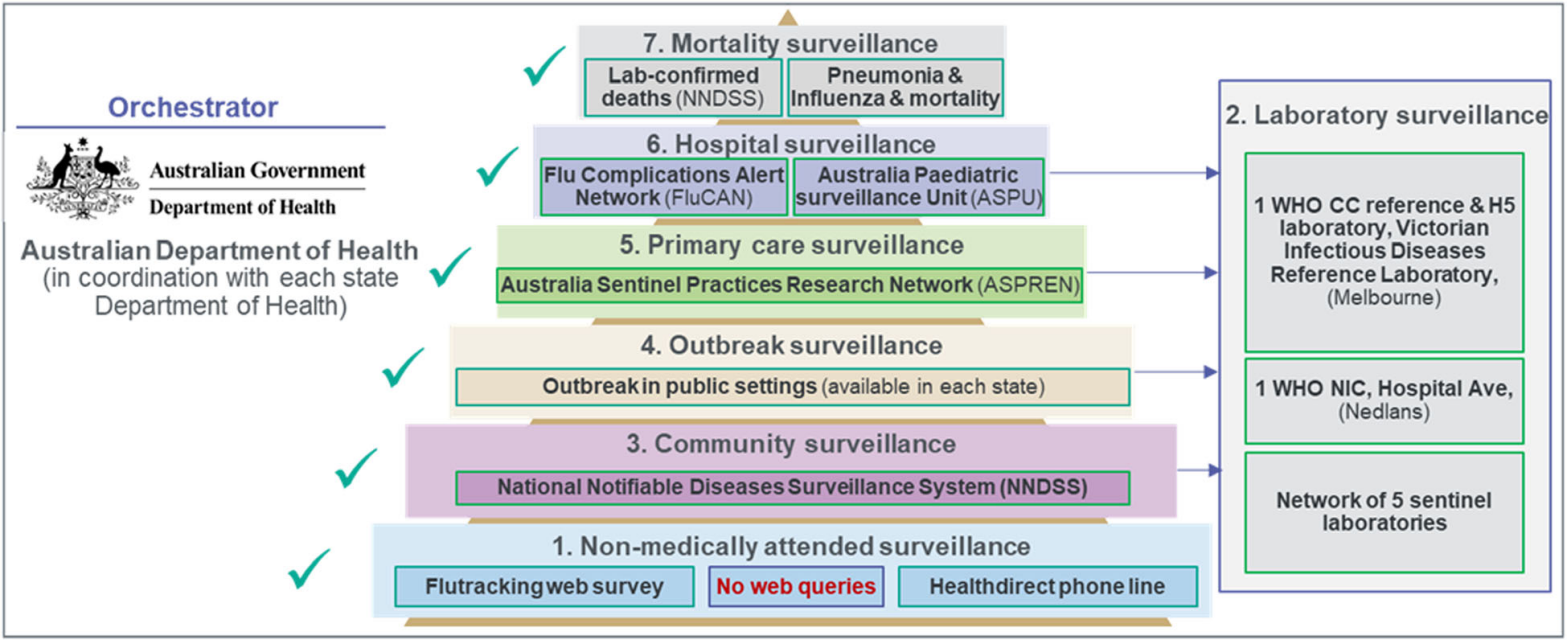
Pyramid of influenza surveillance system in Australia

This country is an historic pillar of WHO influenza surveillance network with a wide scope of surveillance activities implemented across its states in a context of devolved health care system (Fig. 1). The National Influenza Surveillance Scheme (NISS) was created in 1994, and progressively expanded to include data sources used to monitor influenza activity and severity in the community [29]. Unlike Malaysia and China, non-medically attended (sub-system 1) Acute Respiratory Infection (ARI) or Influenza-Like Illness (ILI) cases and healthcare-seeking behaviours were captured through an ongoing web survey (info.flutracking.net) and the medical advice line HealthDirect, respectively [36]. For virological surveillance (sub-system 2), a network of sentinel laboratories across Australian states reported the total number of tests performed each week, and the positive specimens are reported by type and subtype. WHO laboratory surveillance was carried out by two NICs, the Division of Microbiology and Infectious Diseases, PathWest Laboratory Medicine Western Australian Public Health Laboratory in Perth and the Victorian Infectious Diseases Reference Laboratory (VIDRL) in Melbourne The VIDRL is also a WHO Collaborating Centre (CC) laboratory for influenza, providing data to support the decision on strain composition for the influenza vaccine in the Northern and the Southern hemispheres twice a year and feeding GISAID’s EpiFlu database.

As well as a mandatory notification system (sub-system 3) collecting laboratory-confirmed influenza cases and deaths through the National Notifiable Diseases Surveillance System (NNDSS), data were complemented by the sentinel networks of General Practitioners (Sub system 5, Australian Sentinel Practices Research Network, ASPREN) and two hospital surveillance schemes (Sub-system 6, Influenza Complications Alert Network [FluCAN] and Australian Paediatric Surveillance Unit [APSU]). FluCAN covered all hospital and Intensive Care Unit (ICU) admissions across 17 sentinel sites, and the APSU investigated severe influenza cases in children below 15 years of age.

There are differences in surveillance scope across states, e.g. in New South Wales state Emergency Department (ED) visits (Public Health Rapid, Emergency, Disease and Syndromic Surveillance, PHREDSS), ILI outbreaks in close settings (sub system 4), and influenza and pneumonia associated mortality are also monitored (sub-system 7) [30]. In Queensland, institutional outbreaks are monitored, and in Western Australia a sentinel system provides surveillance through primary care with laboratory links.

#### China

**Fig. 2:**
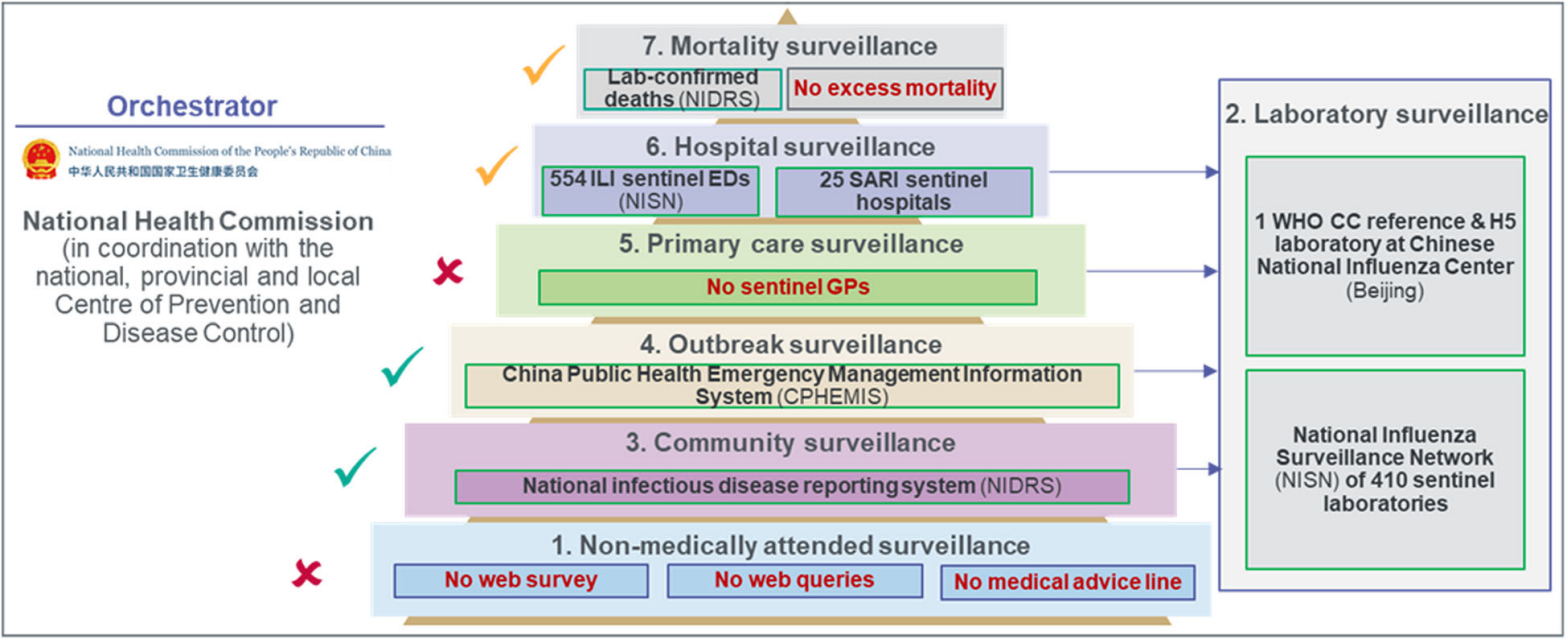
Pyramid of influenza surveillance system in China

China has developed several surveillance tools to cover the impact of seasonal influenza epidemics, but only selected outcomes are made publicly available (Fig. 2). The CNIC has developed an information system with three online components: The Influenza Surveillance Information System, the Infectious Disease Surveillance Platform and the Influenza Prediction and Early Warning Platform. Local Centers for Disease Control and Prevention (CDC) across China can access this system to 1) report cases of ILI, pneumonia of unknown aetiology, and Severe Acute Respiratory Infections (SARI); and 2) upload laboratory testing results [32]. The National Influenza Surveillance Network (NISN) is the surveillance backbone and includes 554 sentinel hospitals (sub-system 5). Each week, 20 samples are collected in each sentinel site and transferred to 410 laboratories, covering all prefecture-level cities and several districts and counties of mainland China. Selected samples from the NISN are processed by the WHO CC laboratory in the CNIC in Beijing (sub-system 2), which then provides the WHO FluNet with influenza type and subtype breakdown, and a phylogenetic and antigenic analysis are sent to GISAID’s EpiFlu database and used for influenza vaccine strain selection and recommendation.

The NISN also played a vital role in the early detection of emerging novel influenza viruses. Built on the existing influenza surveillance network, the CNIC developed a novel influenza identification platform in order to detect zoonotic infections. This system enabled the CNIC to confirm China’s first human infections with A(H7N9), A(H10N8) and A(H5N6) viruses just 1–3 days after receiving specimens. In parallel, the China Public Health Emergency Management Information System (CPHEMIS) reported ILI outbreaks of more than 10 cases within 5 days in all close settings to the local CDC and NIC (sub-system 4). Specimens collected from each outbreak were used to evaluate the aetiology. A mandatory notification system (National Infectious Disease Reporting System [NIDRS]) covered seasonal influenza as a Class C disease, with mandatory reporting of clinically diagnosed and laboratory-confirmed cases in all healthcare settings nationwide (sub-system 3).

Since the H1N1 2009 pandemic, a SARI sentinel network of 25 hospitals is being progressively rolled out across Chinese provinces to monitor hospital admissions, ICU admissions, and deaths caused by influenza (sub-system 6). China is also taking part in the Global Influenza Hospital Surveillance Network supported by the Foundation for Influenza Epidemiology with one contributing site, Fudan University, in Shanghai, supporting better characterisation of viral information and support linkage with clinical characteristics of samples [37]. As well as deaths notified under the NIDRS and published by the CCDC, the National Health Commission (NHC) publishes the main causes of mortality in its China Health Statistical Yearbook, including respiratory deaths, derived from the Disease Surveillance Points (DSP) system from 161 mortality data sources (sub-system 7) [38]. No surveillance tools such as web surveys, phone advice line or search engine queries were used to monitor non-medically attended cases in the community.

#### Malaysia

In Malaysia, influenza surveillance focused mainly on influenza activity and virology.

**Fig. 3:**
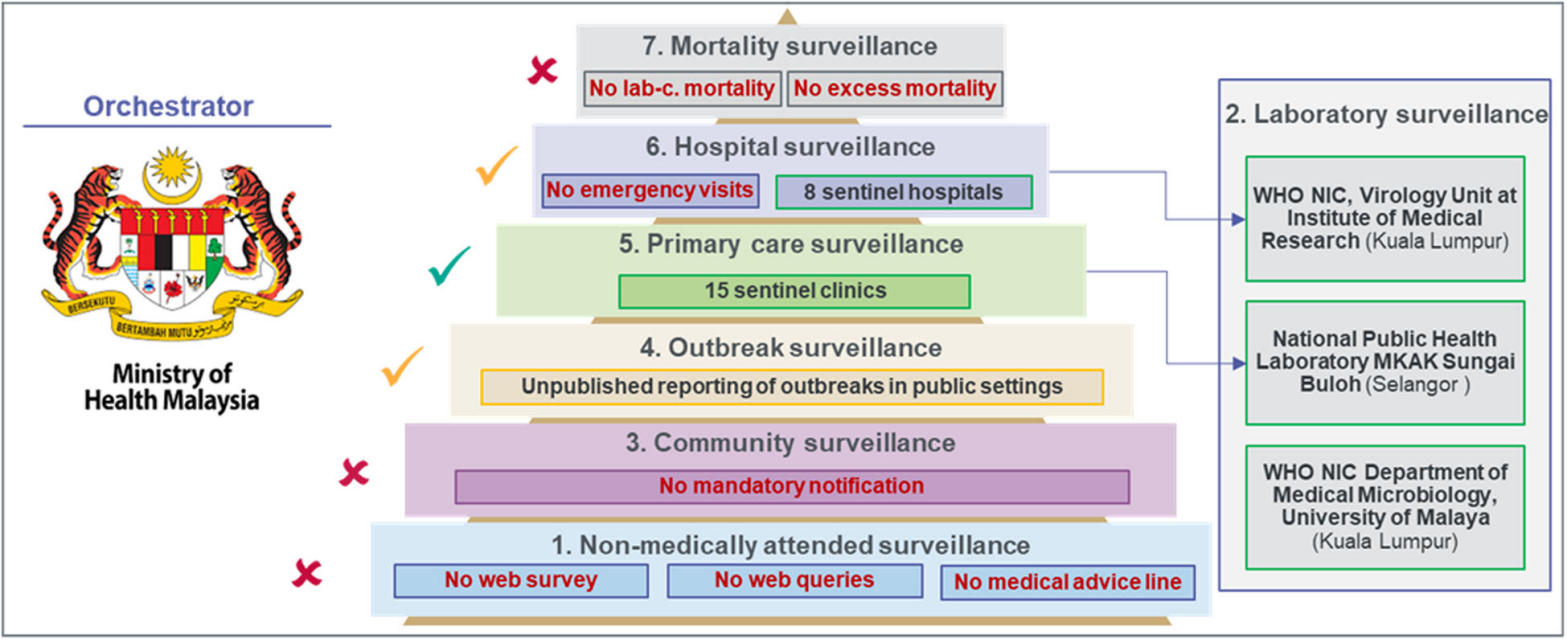
Pyramid of influenza surveillance system in Malaysia

Figure 3). 15 sentinel clinics under ILI syndromic surveillance (sub-system 5) and 8 sentinel hospitals under SARI syndromic surveillance (sub-system 6) collect five samples per week each. Then, specimens are sent to two designated laboratories: the MKAK Sungai Buloh for the ILI specimens and the Virology Unit of Institute of Medical Research (WHO NIC) for SARI specimens (sub-system 2) [34]. As a WHO NIC, the Department of Medical Microbiology in University of Malaya reports isolates separately to a WHO CC and laboratory test results are submitted to WHO FluNet database [9]. Although respiratory virus outbreaks in close settings may be reported to the MOH, there was no reporting obligation as influenza is not a notifiable disease in Malaysia (sub system 4). As well as the data produced by the eight sentinel hospitals monitoring SARI cases such as hospitalizations, ICU admissions and deaths were not monitored or reported. Similarly, data on influenza-related ED visits were not collected for surveillance purposes in Malaysia. No surveillance tools were used to monitor non-medically attended cases occurring in the community.

### Adherence to WHO guidance

Australia, China, and Malaysia contribute to WHO basic requirements in terms of monitoring of influenza virology and activity. All three countries provide the results from their sentinel schemes in primary and secondary care on a weekly basis to their respective NIC, supporting the identification of seasonality patterns and circulating subtypes. Compared to Malaysia, both China and Australia have developed more in-depth virological surveillance and both countries have a WHO CC reference and H5 reference laboratory, providing data on influenza virus genome sequencing, antigenic characterisation, antiviral resistance, and identification of pathogens with pandemic potential such as avian influenza viruses. These contribute to WHO FluNet, the Global Initiative on Sharing Avian Influenza Data (GISAID)‘s EpiFlu database and the biannual WHO vaccine recommendations [9, 39].

Australia and China cover both influenza-related hospitalizations and deaths, as well as respiratory outbreaks occurring in close settings, whilst influenza surveillance in Malaysia provides limited information on these outcomes. By monitoring non-medically attended events, reporting suspected or laboratory-confirmed cases in emergency, routine, and intensive care hospital wards, and estimating influenza and pneumonia-related mortality at the state level, Australia provides a better picture of the burden of influenza.

### Data granularity

In the fortnightly reports from the Australian Department of Health, data on laboratory-confirmed cases from the NNDSS are reported in 5-year age groups, by virus type and sub-type and by state. As well as age, information on underlying conditions is made available for most severe cases, with the severity of the infection documented through data on ICU admission and death. No information on sex and immunisation status are publicly available. In China, data include age and sex, following WHO guidance, but there are no data available on underlying conditions, immunization status, or the complications of influenza, which are usually specifically recommended for SARI surveillance. Although the data reported by the Malaysian MOH are broken down by age, this is not in line with the age stratification recommended by WHO. Also, no information on underlying conditions, immunization status, and consequences of influenza are recorded.

### Data timing

In Australia, influenza surveillance is performed all year, with reports produced every 2 weeks during the influenza season (May–September) and every month during the inter-seasonal period. In China, NISN samples are reported all year to the city, provincial, and national CDC each weekly. In Malaysia data are reported all year on a weekly basis.

### Data representativeness

In Australia, the geographical representativeness is national for the data produced by sentinel GP and hospital schemes and the virological network whilst outbreak surveillance is performed at state level. Population representativeness is ensured by a systematic swabbing of 25% of patients with selected symptoms by sentinel GPs. For excess-mortality modelling, only New South Wales state has formally developed a surveillance of Pneumonia and Influenza-associated deaths. In China, NISN sentinel sites are geographically representative given their presence all 31 provinces, autonomous regions, and municipalities directly under the Central Government. A recent study highlighted weaknesses in coverage and poor reliability of influenza data from the NISN after the H1N1 pandemic across eight provinces (Tibet, Hebei, Ningxia, Shanxi, Qinghai, Xinjiang, Yunnan, and Inner Mongolia) [40]. The SARI sentinel system may not be nationally representative as it only covers 25 provinces out of 35. Furthermore, influenza cases, mostly clinically diagnosed, notified with the NIDRS system show inconsistencies in notified cases reported across Chinese provinces, thus questioning the representativeness of such data. Surveillance sentinel sites are present in all regions and as such geographically representative. The sampling instructions for sentinel sites ensure population representativeness.

### Sampling strategy

In Australia, the ASPREN network recommends sentinel GPs to swab 25% of the patients presenting with fever, cough or fatigue, amounting to 1000–5000 samples per week over a total population of 25 million inhabitants. In China, the NISN sampling strategy varied: in the South, 10–40 samples were collected from each site every week, with an average of 20 samples each week per site, whereas in the North, 20 samples per site were collected per week during the flu season, and 20 samples per site each month at other times. The CCDC processes up to 20,000 samples per week from the NISN over a total population of 1398 million inhabitants. The NIDRS uses clinical diagnosis and a range of test types, many of which are less sensitive than RT-PCR (which is used for NISN). The sampling strategy in Malaysia was five samples per week per site for reverse transcriptase polymerase chain reaction (RT-PCR) testing, but at the discretion of the sentinel sites. Only up to 100 samples per week were reported due to the limited number of sentinel sites for a population of 39 million inhabitants in 2019.

### Data communication

The Australian Department of Health publishes the national influenza surveillance report fortnightly during the season, and monthly otherwise [28]. In China, the CNIC has generated online weekly influenza reports since 2005 to share when, where, and which influenza viruses were circulating using NISN and CPHEMIS surveillance data. The weekly reports, in both Chinese and English, are emailed to key stakeholders and are also made available on the CNIC website [32]. The NIDRS notified influenza cases and deaths are shared with a breakdown by age and province on a monthly basis by the CCDC on the Public Health Science data platform [31]. Hospital admission data are not available, except occasionally in scientific publications from observational studies [41]. Although laboratory-confirmed influenza cases by subtype are available through the WHO FluNet website, weekly and annual influenza surveillance reports are not made publicly available by the Malaysia MOH.

### Expert recommendations

Australia already has a detailed surveillance scheme and key elements to monitor and estimate the burden of influenza. Nevertheless, it would benefit from better links between surveillance sub-systems, e.g. community and hospital data, to analyse trends in severity or better anticipate hospital capacity. Additionally, formalising ad hoc absentee surveillance could further document the broader impact of influenza. Finally, using excess mortality modelling at a national level, as currently performed in Europe with EuroMOMO or in the US with the US-CDC method, could complement the death notification system [42, 43].

In China, influenza epidemiology outcomes on a longitudinal and nationwide perspective would be useful to estimate the national burden of influenza with the absolute number of influenza cases, consultations, hospitals admissions, and deaths. Estimates of influenza incidence, associated hospitalization rate, and mortality rate could be produced on an annual basis at a minimum by province. It is also suggested to expand the current SARI network to cover all provinces and publish data on severe influenza cases using the existing weekly bulletin from the CNIC. To compensate for the limitations of influenza deaths notified under the NIDRS, the adoption of an excess-mortality model to estimate the mortality attributable to influenza or for all causes, as previously performed by Chinese research groups at national, provincial, and city levels would be useful [40, 44–46]. Finally, the use of surveillance tools to monitor non-medically attended events could be considered, so as to best anticipate the evolution of the epidemic, such as self-reported web-survey, medical phone line surveillance or monitoring of web queries.

In Malaysia, the activity and burden of influenza are acknowledged by the health authorities and medical community. The Malaysian surveillance system is designed to detect outbreaks rather than to be representative and exhaustive in measuring the public health burden and economic impact of influenza. The main challenge is to prioritize respiratory infections and influenza in the public health agenda and provide more resources to influenza prevention programmes in a context of competing budgetary needs with other health priorities. Reliable data, from an enhanced national influenza surveillance system, are needed for policy decisions to effectively prevent influenza, especially among the high-risk groups. For example, an expansion of sentinel networks in primary and secondary care and the broader use of RT-PCR for the confirmation of cases would help to better characterise the influenza burden and circulation of different subtypes. Also, better granularity is needed, with an age stratification following WHO guidance [i.e. 0 to < 2 years, 2 to < 5 years, 5 to < 15 years, 15 to < 50 years, 50 to < 65 years, ≥ 65 years] and information on sex, underlying status, and immunisation status [22]. The official health authorities should be encouraged to publish influenza epidemiology and virology reports on weekly and annual basis. These could be complemented by annualized disease burden estimates to be communicated with the lay public through official channels and multi-stakeholder initiatives such as the Immunize4life [47].

## Discussion

This framework has allowed influenza surveillance systems from three WPR countries in distinct influenza transmission zones to be compared and adherence to WHO guidelines to be assessed. Given the differences in surveillance systems in terms of structure and targeted outcomes, there was considerable variability across the three countries, allowing the identification of gaps which were highlighted in the expert recommendations. Australia demonstrated the widest scope of surveillance, covering all sub-systems and most outcomes, followed by China and then Malaysia [22]. Malaysia, where weekly and annual influenza reports with breakdown of data per age, gender and risk condition were not publicly available, was less advanced than China and Australia in terms of data granularity and data communication. Data representativeness varied considerably across the three countries, and in China and Malaysia there was often no information on the proportion of the population covered by sentinel schemes making not possible to interpret a comparison of reported specimens between countries. On the contrary, data timing was aligned, with all three countries implementing all year long surveillance with reporting on a weekly basis. These differences in terms of breadth and depth of influenza surveillance systems should be interpreted in the light of the objectives followed by each country [48]. Australia has developed a sophisticated laboratory surveillance, and now aims at comprehensively measuring the burden of influenza and assess the impact of its national immunization programme. In China, the willingness to develop an early warning system to prevent potential pandemic has supported the adoption of Beijing NIC as a WHO CC and H5 reference laboratory and driven the expansion of the Early Warning System, NISN, and CPHEMIS recently, although there are still wide disparities across Chinese provinces. Moreover, mandatory notification tools such as the NIDRS, that records the number of influenza cases and deaths across China, may have represented a conservative estimate of the overall influenza burden (e.g. in 2018 only 765,186 cases and 153 deaths were notified). In Malaysia, beyond WHO basic requirements, the 2018 influenza surveillance protocol sets out the objectives pursued, including: “To provide data that can contribute to the estimation of the burden of severe respiratory disease associated with influenza and other respiratory pathogens”, which is yet to be fulfilled [34].

The novelty of this study is to provide a first cross-country comparison and evaluation of influenza surveillance system in the Western Pacific Region with prioritized recommendations from the APACI network experts. Although WHO resources provide clear guidelines on influenza surveillance, there are no published comparative analyses of influenza surveillance systems in the region yet, as performed by the WHO EURO and ECDC region for instance [49]. The contextual elements of influenza surveillance are important when aiming to share and compare information of the burden of influenza between countries. So far, existing published literature on influenza surveillance tends to focus on a single country, specific seasons and provide no comparative baseline for discussion.

Overall, to broaden the understanding of influenza burden in the WPR and enhance surveillance data representativeness, it is important to improve the detection capacity of influenza cases. This could be achieved by a more systematic testing of ILI, ARI and SARI cases at community and secondary care levels. In addition, the use of rapid PCR tests at healthcare sites which have demonstrated high specificity could help to better detect laboratory-confirmed influenza cases, and support a better care management of patients at a hospital level [50].

Finally, a cluster detection with an early warning system and “emergency” validation to detect new virus or variants linked with clinical severity is of particular importance [51]. China recommends this whenever for more than 10 cases, which is an interesting approach to be further developed under the influenza pandemic preparedness framework repurposed for emerging viruses.

In addition to monitoring the impact of influenza epidemics through the different surveillance tools described in this study, monitoring VCR in target groups and real-world vaccine effectiveness allows the impact of immunisation programmes on influenza circulation to be measured [17, 18, 19]. Collecting data on immunisation status through registries or patient records is important, but such technological infrastructure is rarely available. In many WPR countries, influenza VCR are documented only in some of the recommended populations and disproportionately rely on influenza sales rather than data from a nationwide adult vaccination registry.

Established influenza surveillance systems are an integral part of pandemic preparedness plans, and as advised by the WHO can be an effective, cost-efficient, and sustainable solution when repurposed to monitor new pathogens, such as coronavirus, as recently proven for the COVID-19 pandemic [52–54]. The results from this study may support improvement of influenza surveillance systems, relying on expert recommendations for consideration by relevant international, national, and sub-national health officials and ultimately support the decrease of the incidence of seasonal influenza. A limitation of this analysis is the inclusion of only three WPR countries, from which the extrapolation to other countries in the region has not been evaluated.

## Conclusions

This study provided an example of how a comparative framework inspired from WHO standard surveillance guidelines can support expert recommendations to drive improvement of influenza surveillance capabilities. Expert recommendations addressed key elements to build stronger surveillance systems such as expanding existing sentinel schemes in primary and secondary care, introducing new tools such as excess-mortality modelling and making surveillance data publicly available on a weekly and annual basis. Such improvements could contribute to better inform national influenza control strategies, enhance public disease awareness, and ultimately improve VCR. Researchers are thus encouraged to use this framework in additional geographies, and further fine-tune it as needed.

## Supplementary Information

**Additional file 1.** Influenza surveillance system overview in Australia, China and Malaysia. Overview and detailed assessment of each influenza surveillance sub-system across the 5 criteria and sub-criteria: 1. Non-medically attended community surveillance, 2. Virological surveillance, 3. Community surveillance, 4. Outbreak surveillance, 5. Primary care syndromic surveillance, 6. Hospital syndromic surveillance & 7. Mortality surveillance

## Data Availability

All data generated or analysed during this study are included in this published article and its additional information files.

## Abbreviations

APACI: Asia-Pacific Alliance for the Control of Influenza
APSU: Australian Paediatric Surveillance Unit
ARI: Acute Respiratory Infection
ASPREN: Australian Sentinel Practices Research Network
AU: Australia
CH: China
CCDC: Chinese Center For Disease Control and Prevention
CDC: Center of Disease Control and Prevention
CNIC: Chinese National Influenza Center
CPHEMIS: China Public Health Emergency Management Information System
DoH: Department of Health
DSP: Disease Surveillance Points
ED: Emergency Department
FluCAN: The Influenza Complications Alert Network
GISAID: Global Initiative on Sharing Avian Influenza Data
GISRS: Global Influenza Surveillance and Response System
GP: General Practitioner
ICU: Intensive Care Unit
ILI: Influenza-Like Illness
MIWG: Chair Malaysia Influenza Working Group
MOH: Ministry of Health
MY: Malaysia
NHC: National Health Commission
NIC: National Influenza Centre
NIDRS: National Infectious Disease Reporting System
NISN: National Influenza Surveillance Network
NISS: National Influenza Surveillance Scheme
NNDSS: National Notifiable Diseases Surveillance System
NSW: New South Wales
PHREDSS: Public Health Rapid, Emergency, Disease and Syndromic Surveillance
RT-PCR: Reverse Transcriptase Polymerase Chain Reaction
SARI: Severe Acute Respiratory Infection
VCR: Vaccine Coverage Rates
VIDRL: Victorian Infectious Diseases Reference Laboratory
WHO: World Health Organization
WHO CC: World Health Organization Collaborating Centre
